# Significance of t (8: 14) in CLL?

**DOI:** 10.4103/0971-6866.69374

**Published:** 2010

**Authors:** K. C. Lakshmaiah, S. Tejinder, Prasanna Kumari, Mangala M. Gowri, C. T. Satheesh, D. Lokanatha, Abraham L. Jacob

**Affiliations:** Department of Medical Oncology, Kidwai Memorial Institute of Oncology, Bangalore – 560 030, India; 1 Department of Pathology, Kidwai Memorial Institute of Oncology, Bangalore – 560 030, India

Sir,

The Burkitt’s lymphoma-type chromosomal translocations such as t (8; 14), t (8; 22) or t (2; 8) are rarely seen in chronic lymphocytic leukemia (CLL) and their significance remains poorly understood. On reviewing the literature, t (8; 14) and t (8; 22) were reported in eight CLL cases each; and t (2; 8) has been reported in two CLL cases.[1]

**Figure 1:**
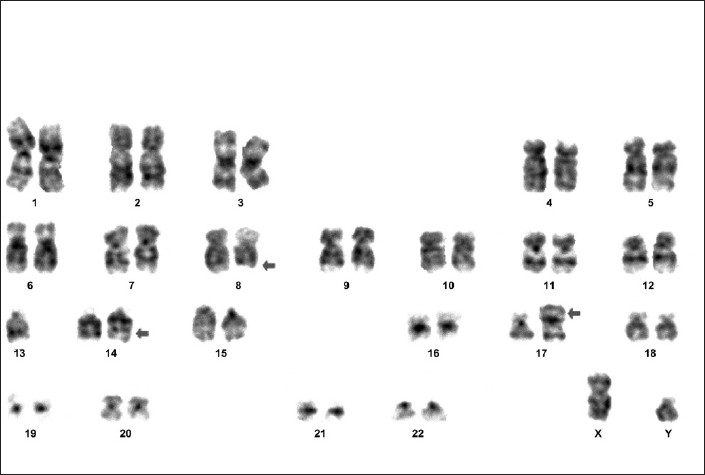
GTG-banded metaphases showing 45, XY, t (8;14)(q24;q32),-13, add (17)(p11)/46, XY

A 65-year-old male presented with leukocytes, in October 2003, at the time of the routine check-up for weakness and fatigability. Peripheral blood cell count showed WBC: 82.6 × 10^9^/l, Hb: 8.1 g/dl, platelets: 93 × 10^9^/l with mature lymphocytes, which accounted for 86% of the differential. The bone marrow aspirates (BMA) were hypercellular with 63% of morphologically mature lymphocytes. Immunophenotyping revealed that such cells were positive for monotypic Igĸ light chain, CD5, CD19, CD20, CD23 and negative for CD10 and FMC7. Cytogenetic analysis performed on BMA showed normal karyotype. Physical examination revealed bilateral cervical lymphadenopathy and ultrasonogram of abdomen showed no hepatosplenomegaly. A diagnosis of CLL was made and according to modified Rai classification, his stage was IV (high risk). The patient was given chlorambucil orally and he obtained complete remission about 12 months later. He was on regular follow up after that, when in August 2007, he came with complaints of generalized lymphadenopathy and splenomegaly. Peripheral blood cell count showed WBC: 68.6 × 109/l, Hb: 9.1 g/dl, platelets: 88 × 10^9^/l with mature lymphocytes, which accounted for 80% of the differential. BMA was hypercellular with 73% of morphologically mature lymphocytes. ZAP70 and CD38 were negative. Cytogenetic analysis revealed 45, XY, t(8;14)(q24;q32),-13, add (17)(p11)/46,XY [Figure 1] (ISCN-2005). Patient was restarted on chlorambucil and achieved remission in 8 months. He is presently doing well and on a regular follow-up.

The rarity of t (8; 14) in CLL our patient persuaded us to explore the literature. After our case, there are now nine cases of t (8; 14) reported in literature. Only one case had no additional or complex karyotypic anomalies and one case transformed to Burkitt’s leukemia. Occurrence of this abnormality in CLL is rare, and the significance of this is unclear. Our patient is doing well in spite of new cytogenetic anomaly detected during relapse, which may be explained that other factors like ZAP70 and CD38 were negative and are related with poor prognosis.[2,3] Further studies of similar cases may shed additional insight into Burkitt’s lymphoma-type chromosomal translocations.
